# Supplementary material for: Cystoseira compressa and Ericaria mediterranea: Effective Bioindicators for Heavy- and Semi-Metal Monitoring in Marine Environments with Rocky Substrates
Source: Plants (Basel). 2024 Feb 15;13(4):530. doi: 10.3390/plants13040530 (PMC10893443; doi:10.3390/plants13040530)
Supplement: Supplementary file 1 [file plants-13-00530-s001.zip › PCA_contributions.pdf]

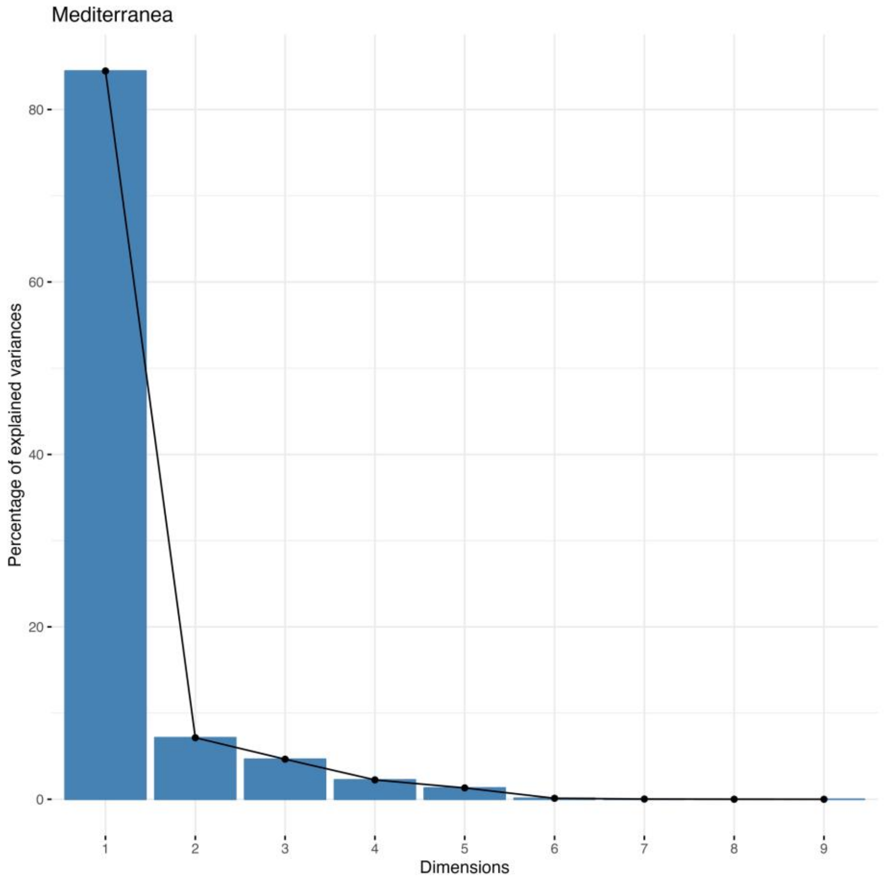

Figure S1. Percentage of explained variance across the different dimensions in the calculated PCA for *E. mediterranea*.

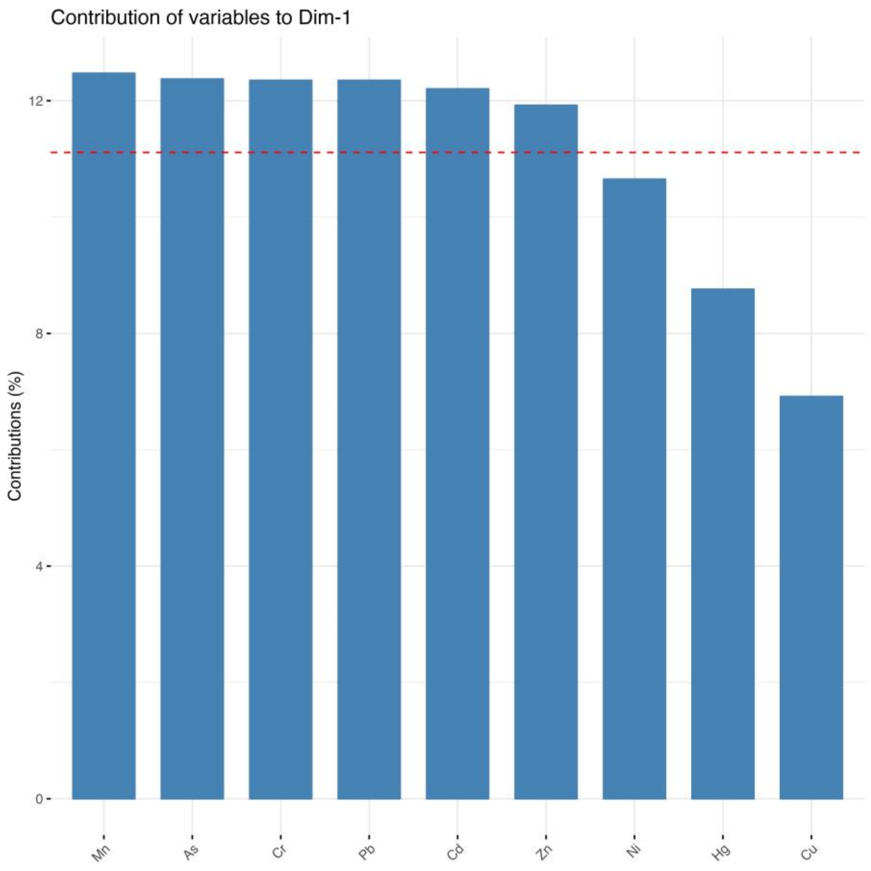

Figure S2. Contribution of heavy- and semi-metals to the first dimension in the calculated PCA for *E. mediterranea*.

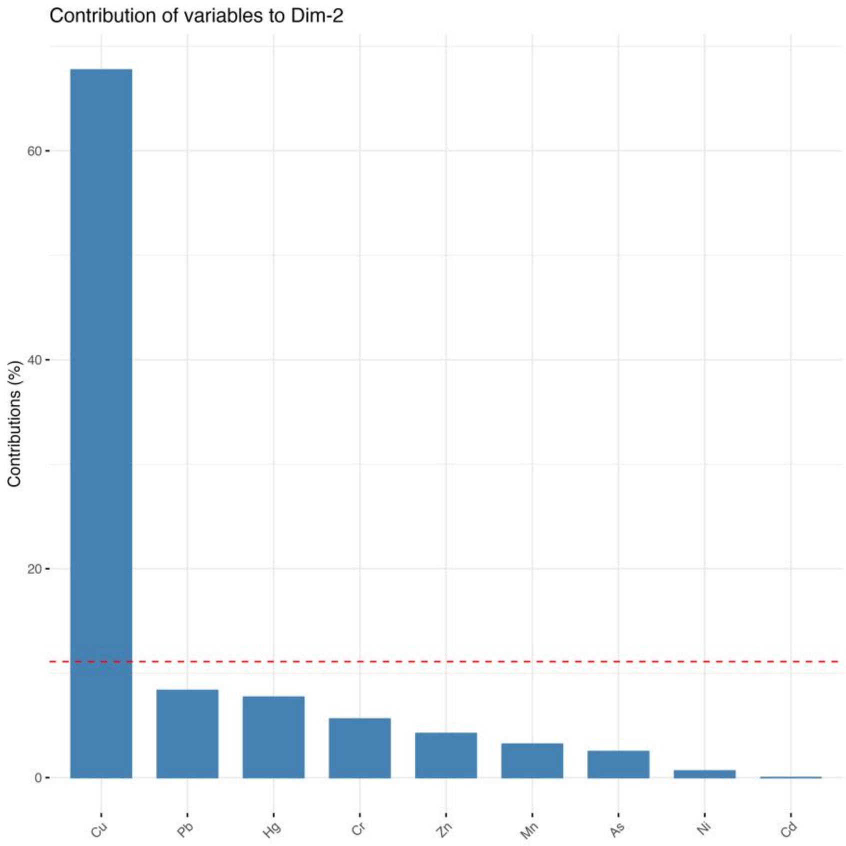

Figure S3. Contribution of heavy- and semi-metals to the second dimension in the calculated PCA for *E. mediterranea*.

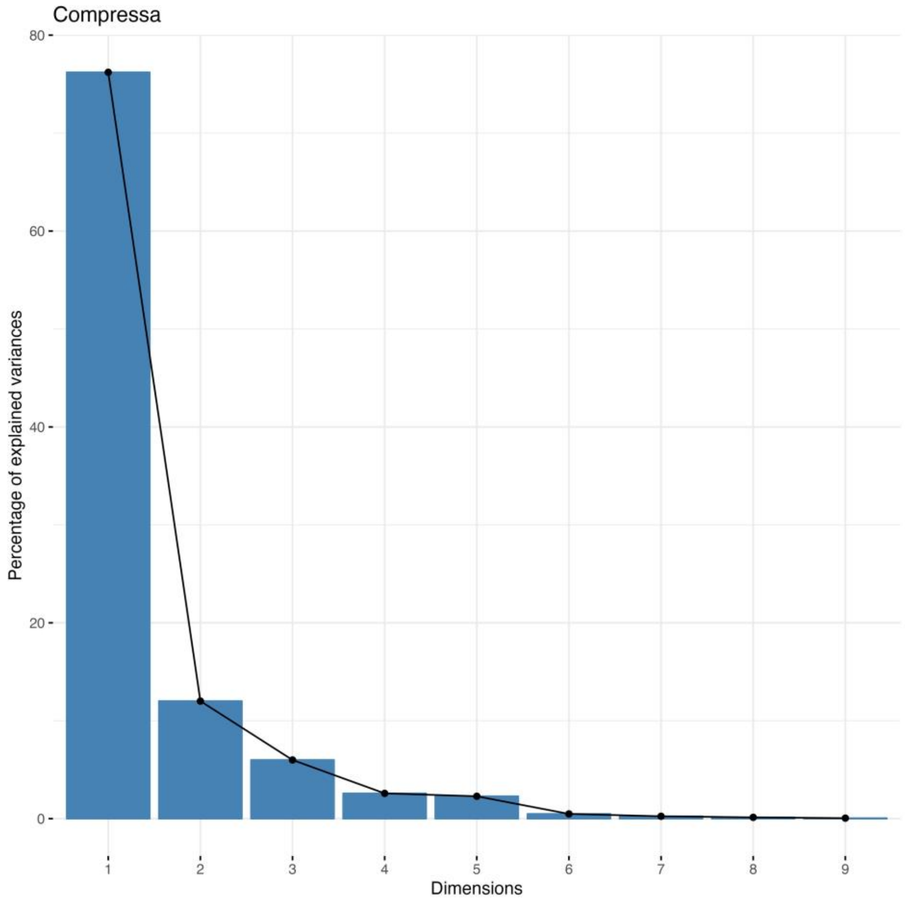

Figure S4: Percentage of explained variance across the different dimensions in the calculated PCA for *C. compressa*.

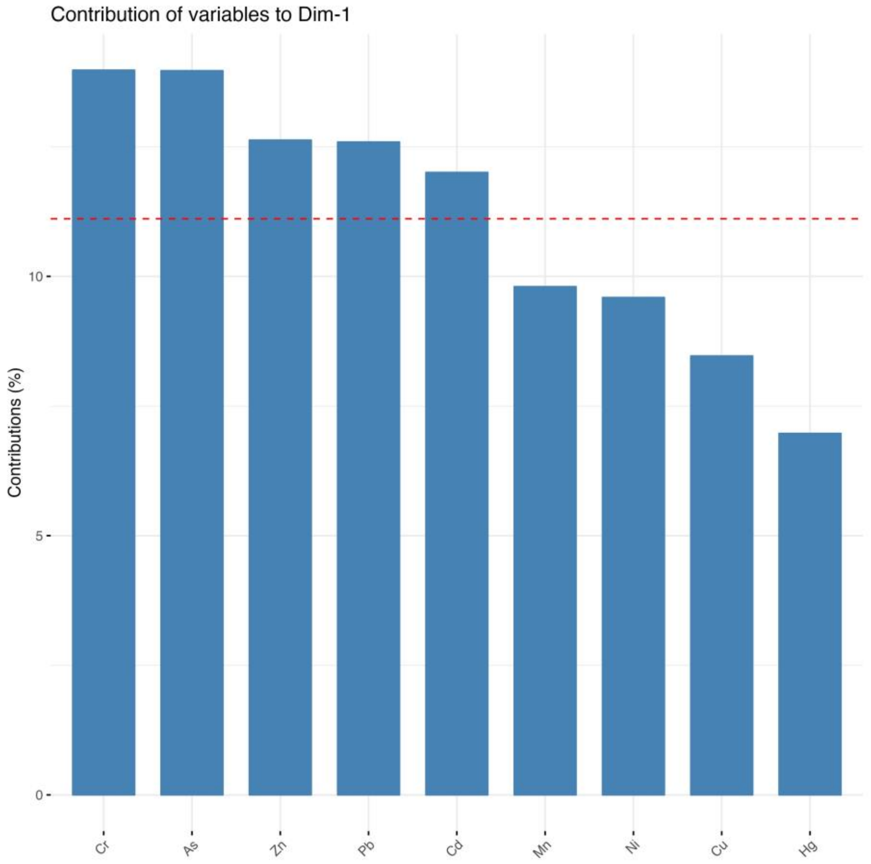

Figure S5. Contribution of heavy- and semi-metals to the first dimension in the calculated PCA for *C. compressa*.

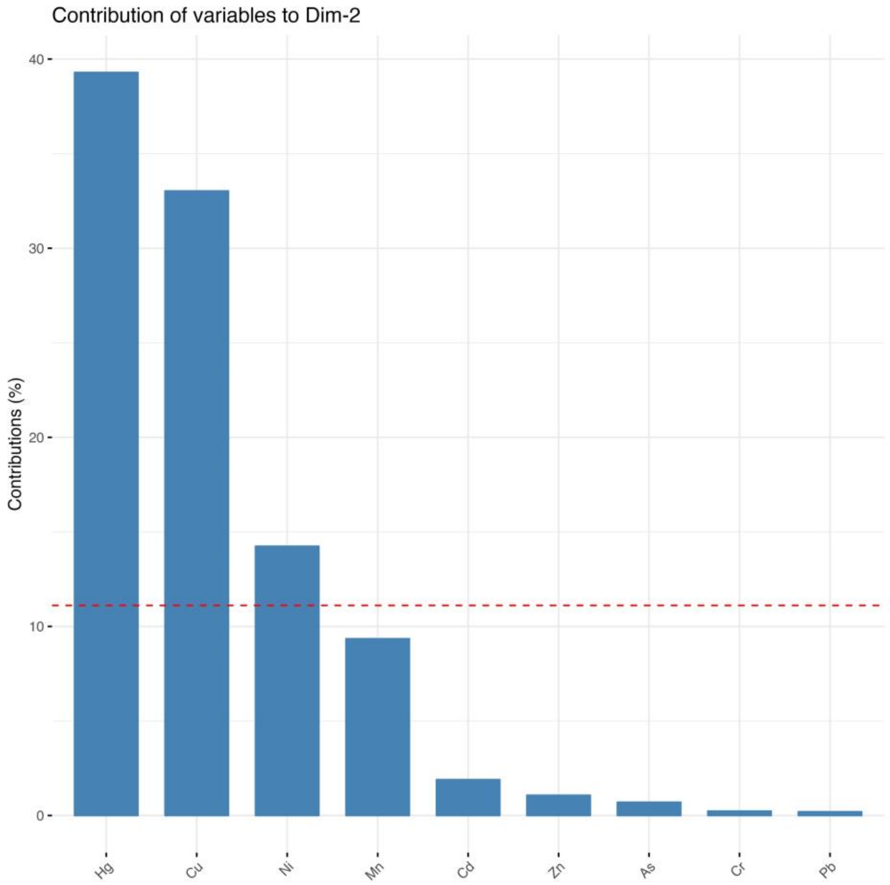

Figure S6. Contribution of heavy- and semi-metals to the second dimension in the calculated PCA for *C. compressa*.
